# Relationship between periodontal disease and preterm low birth weight: systematic review

**DOI:** 10.11604/pamj.2016.24.215.8727

**Published:** 2016-07-12

**Authors:** Amare Teshome, Asmare Yitayeh

**Affiliations:** 1 Department of Dentistry, School of Medicine, College of Medicine and Health Sciences, University of Gondar, Ethiopia; 2 Department of Physiotherapy, School of Medicine, College of Medicine and Health Sciences, University of Gondar, Ethiopia

**Keywords:** Low birth weight, periodontal disease, Preterm birth, systematic review

## Abstract

**Introduction:**

Periodontal disease is a neglected bacterial infection that causes destruction of the periodontium in pregnant women. Yet its impact on the occurrence of adverse pregnancy outcomes has not systematically evaluated and there is no clear statement on the relationship between periodontal disease and preterm low birth weight. The objective of this study was to summarize the evidence on the impact of periodontal disease on preterm low birth weight.

**Methods:**

We searched the following data bases from January 2005 to December 2015: CINAHL (cumulative index to nursing and allied health literature), MEDLINE, AMED, EMBASE (excerpta medica database), Cochrane library and Google scholar. Only case-control studies with full text in English were eligible. Critical appraisal of the identified articles was done by two authors independently to provide the possible relevance of the papers for inclusion in the review process. The selected Case control studies were critically appraised with 12 items structured checklist adapted from national institute of health (NIH). Odds ratio (OR) or risk ratios (RR) were extracted from the selected studies. The two reviewers who selected the appropriate studies also extracted the data and evaluated the risk of bias.

**Results:**

Of 229 articles, ten studies with a total of 2423 participants with a mean age ranged from 13 to 49 years were met the inclusion criteria. The studies focused on preterm birth, low birth weight and /or preterm low birth weight and periodontitis. Of the selected studies, 9 implied an association between periodontal disease and increased risk of preterm birth, low birth weight and /or preterm low birth weight outcome (ORs ranging from 2.04 to 4.19) and only one study found no evidence of association.

**Conclusion:**

Periodontal disease may be one of the possible risk factor for preterm low birth weight infant. However, more precise studies with randomized clinical trial with sufficient follow-up period must be done to confirm the association.

## Introduction

Periodontal diseases are bacterial infections of the tooth supporting structures, which causes inflammation and destruction of the periodontium. This bacterial infections are adhered to the periodontal tissue by biofilm, which is a complex structure of bacteria and discernable by the excretion of a protective and adhesive matrix [1]. The progression of bacterial infection leads the periodontium to become severely destructed and causes a chronic and systemic challenge with bacterial substance and host-derived inflammatory mediators are capable of initiating and promoting systemic diseases [2]. Oral health and its relationship to systemic health is a global health concern due to 90% of the population is affected by periodontal disease- either gingivitis or periodontitis [3]. There is emerging evidence that suggested periodontal disease is associated with cardiac disease, diabetes mellitus, respiratory infection and adverse pregnancy outcomes [4]. The prevalence of Periodontitis is high in pregnant mothers (40%) [5], and all these mothers with periodontitis have seven times at risk of having preterm or low birth weight baby [6]. The hormonal changes during pregnancy, promotes inflammatory response that facilitates the occurrence of periodontal disease. Due to the change in hormonal level 50-70% of women develop gingivitis during their pregnancy. The increased level of progesterone and estrogen in plasma during pregnancy can affect periodontal structure through interference in sub gingival micro flora composition, maternal immune system, and facilitates pro-inflammatory mediator production [7]. Preterm birth (PTB) and Low birth weight (LBW) are considered a primary public health challenge and the most relevant biological determinant of new-borns' survival, both in developed and in developing countries. They have a marked effect or influence on both the health care system and the individual families. This necessitates the uninterrupted search for risk factors for preterm birth and LBW that are amenable to prevention [8]. The importance of preterm birth and LBW not only comes from its capacity to predict increased risk of mortality and morbidity among infants born with this condition but it also reflects the mother's exposure to other risk factors such as unfavourable socio-economic conditions, malnutrition and diseases of the mother, among others [9]. A case-control study done by Offenbacher et al in 1996 showed that, periodontal disease is a significant risk factor for preterm low birth weight with odds ratio of 7.9 [6]. Other similar study done by Hill (1998) found that periodontal bacteria's have the potential to produce infection in the upper genital tract in pregnant women, causing preterm birth. Hill also found bacterial species of Fusobacterium nucleatum and Capnocytophaga in the amniotic fluid cultures in women with preterm labor [10]. A randomized controlled trial done by lopez et al [11] showed that periodontal therapy reduced the incidence of preterm and low birth weight in women with periodontal disease. However, Michalowicz et al revealed that periodontal therapy had no effect on the incidence of preterm birth [12]. The past 10 years have witnessed in an increase in research to explore the association between periodontal disease and adverse pregnancy outcomes. Some of the previous studies have found a significant relationship between preterm birth and periodontal disease [13–15]. However, there are evidences which disapprove the adverse effects of Periodontitis on pregnant women [16, 17]. Due to this inconsistency between different studies on the association between periodontal and preterm low birth weight, a confirmation of periodontal disease as a possible risk factor for this adverse effect would be of great public health problem due to its preventive and curable nature of the disease. Synthesising evidence on the impact of periodontal disease on adverse pregnancy outcomes (preterm low birth weight) can help to develop public health awareness and inform the health system to implement relevant measures and dental follow-up of pregnant mother during their ANC follow-up time, to define the role of each health professionals on pregnant mother. Our objectives were to summarize the evidence on the impact of periodontal disease on preterm low birth weight.

## Methods

**Searching strategy and inclusion:** An electronic database search for relevant case- controlled studies published in English (as translation funding was not available) was conducted from February 2015 to September 2015 on the following databases: Cumulative Index to Nursing and Allied Health Literature (CINAHL), MEDLINE, Allied and Complementary Medicine (AMED), EMBASE, Cochrane library and Google scholar. The search terms used were gingivitis, periodontal disease, low birth weight, Oral health, and preterm delivery. To identify relevant article, titles and abstracts of retrieved papers were exported to Endnote where duplicates were identified and removed by one reviewer (AT). If needed full texts of peer-reviewed relevant articles were retrieved, assessed and their reference lists were hand searched to identify further relevant studies.

**Selection of studies:** The method segment of listed case- controlled studies was extracted and two reviewers (AT, AY) employed the pre-determined inclusion criteria to screen for relevant full text case- controlled studies. Both the reviewers were blinded to journal, authors, and results. There were no any conflicts between the two reviewers in final selection decisions. Studies were included for data extraction and analyses, if they met the following predetermined inclusion criteria.

**Inclusion criteria :** Articles were included in this systematic review if they full field the following criteria

**Study type:** Full text case control articles written in english which are recently published (since 2000) in peer reviewed journals, primary journals, be on human subjects

**Participants:** Females of the reproductive age group.

**Figure 1:**
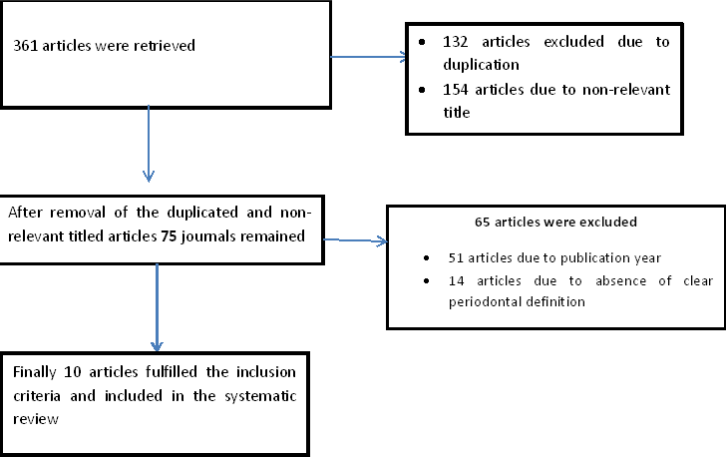
Flow chart showing the article selection process

**Operational definition of periodontitis and outcome measures:** Preterm birth (PTB): delivery at fewer than 37 weeks of gestation. Low birth weight (LBW): delivery of an infant with a birth weight of less than 2.5 kg (Figure 1).

**Quality assessment:** The selected case-control studies were critically appraised with 12 items structured checklist adapted from the national institute of health (NIH). All articles were evaluated based on 12 points of the checklist and the quality of the articles were given as very good, goo and poor if the articles got more than 8, between 5 and 8 and less than 5 points respectively. The checklist assesses the methodological quality of articles based on important criteria, such as: appropriateness of research questions or objective, clarity of definitions on cases, clearly stated definition, inclusion and exclusion criteria, randomization of the cases and controls, measures of exposure/ risk, blindness of evaluator, and motioned and controlled cofounders (Table 1).

**Table 1:** Quality assessment result of the included case control studies (NIH)

NO	Criteria’s	Article 1	Article 2	Article 3	Article 4	Article 5	article 6	article 7	Article 8	Article 9	Article 10
1	clearly stated objective	Yes	Yes	Yes	Yes	Yes	Yes	Yes	Yes	Yes	Yes
2	defined study population	Yes	Yes	Yes	Yes	?	Yes	Yes	Yes	Yes	Yes
3	Sample size justification	Yes	?	Yes	Yes	No	No	Ye	No	No	Yes
4	Homogenous cases and controls	Yes	Yes	Yes	Yes	Yes	yes	Yes	Yes	Yes	Yes
5	inclusion and exclusion criteria	Yes	Yes	Yes	Yes	Yes	yes	Yes	Yes	Yes	Yes
6	differentiated cases from controls	Yes	Yes	Yes	Yes	Yes	Yes	Yes	Yes	Yes	Yes
7	Random selection	?	Yes	Yes	Yes	No	No	No	Yes	Yes	Yes
8	concurrent controls	Yes	Yes	Yes	Yes	Yes	Yes	Yes	Yes	Yes	Yes
9	Baseline assessment	Yes	Yes	?	Yes	No	Yes	No	No	No	Yes
10	measures of exposure	Yes	?	Yes	Yes	Yes	Yes	Yes	Yes	No	yes
11	assessor blindness	Yes	Yes	No	Yes	Yes	Yes	Yes	No	Yes	Yes
12	Controlled confounding factors	Yes	No	Yes	No	Yes	Yes	Yes	No	yes	Yes
Total		11	9	10	10	9	10	10	8	9	12

**Data extraction:** The following data were extracted from the included case-control studies: outcome measures (RR/OR), and follow-up period, and study year, smoking, and alcohol used. The operational definition of periodontitis was extrapolated from the studies and reported. These data were then compiled into a standard table. The two reviewers (AT, AY), who selected the appropriate studies also extracted the data and evaluated the risk of bias.

## Results

**Search yield:** The search from the databases and manual search from Google retrieved 229 articles, after removal of duplicates. After screening title, abstracts and references 154 papers were removed. Full text was obtained for 75 papers. Of which, 65 papers were eliminated as they did not meet inclusion criteria and finally, a total of 10 articles were considered for the systematic review (Figure 1). The studies were conducted in seven countries: 3 articles in India, 2 articles in Brazil, and one each from Iran, Argentina, Jordan, Senegal, and Tanzania each (Table 2, Table 3).

**Table 2:** Characteristics of the included studies in the systematic review

Author/publication year	Country	Sample size case and control	Definition of Periodontitis	Confounders controlled	Outcomes OR/RR(95% CI)	Conclusion
Ahmed Haerian et al. 2013 [18]	Iran	Case =44 Control=44	CPTIN	Yes	Mothers of LBW infants (p=0.042), and more deep pockets (p=0.0006,	Periodontitis is a potential risk factor for LBW
Cruza et al. 2005 [19]	Brazil	Case =102 Control =200	CAL>4mmat least four teeth	Yes	OR=2.15; 95% CI: 1.32-3.48), low birth weight	Periodontal disease is a possible risk factor for low birth weight
Cisse et al, 2015 [20]	Senegal	Case=129 Control =258	CAL≥ 3 mm in at least 2 sites and PD≥ 4 mm	yes	OR = 4 [2.3 - 5.7]	Periodontitis was significantly associated with low birth weight
Moliterno et al, 2005 [21]	Brazil	Case=76 Control=75	PPD>4 least four sites and CAL>3 mm,	Yes	5 3.48, 95% (CI): 1.17; 10.36	Periodontitis was considered a risk indicator for LBW
Mannem and Chaval,2011 [22]	India	Case=52 Control=52	CAL≥3 in at least four teeth	Yes	Odds ratio=137.5, p<0.0001	Periodontal disease could be a risk factor for preterm labor
Grandi et al , 2010 [23]	Argentina	Case=53 Control=79	bleeding index of 0-3index CAL > 1 mm and > 30% of sites involved		AOR=4.19; 95% CI: 1.28 – 13.69, p = 0.018for bleeding index AOR=5.14; 95% CI: 1.50 – 17.6 Pocket depth	bleeding index and periodontal pocket depth are risk factors for preterm birth

**Table 3:** Characteristics of the included studies in the systematic review

Author/publication year	Country	Sample size	Definition of periodontitis	Confounding factors	Outcomes OR/RR(95%CI)	Conclusion
Grandi et al , 2010 [23]	Argentina	Case=53 Control=79	bleeding index of 0-3index CAL > 1 mm and > 30% of sites involved		AOR=4.19; 95% CI: 1.28 - 13.69, p = 0.018for bleedingindex AOR=5.14; 95% CI: 1.50 - 17.6 Pocket depth	bleeding index and periodontal pocket depth are risk factors for preterm birth
Kukkamal et al 2014 [24]	India	Case=100 Control=100	PPD≥4mm 60%=7-9mm	Yes	(p<0.001). (x^2^ 92.8, p<0.001). (60% vs. 3%; x2 97.9, p<0.001)	There was significant co-relation between Periodontitis and Preterm birth and Low Birth Weight.
Khader et al 2009 [25]	Jordan	Case =148 Control=438	CAL>3 mm and PPD >3 mm	Yes	2.04 (95%Ci: 1.59, 2.61) For 1 mm depth increase and 1 mm increase in CAL 2.04 (95%CI: 1.59, 2.61)	The extent and severity of periodontal diseases appeared to be associated with increased odds of PLBW delivery
Smitha. K, et al, 2013 [26]	India	Case=50 Control=50	CAL>3mm	Yes	adjusted odds ratio of 3.16	periodontitis is significantly associated with PTLBW
Mumghamba and Manji, 2007 [27]	Tanzania	Case= 150 Control = 223	PPD>4mm or bleeding 30% 0f examined surface.	Not listed.		No significant association was found between Periodontitis and preterm birth

**Quality:** The quality assessment scores and the decisions of each item for the included case control studies are shown in Table 1.

**Characteristics of included studies:** The characteristics of the 10 case-controlled studies are listed in Table 2, Table 3. All included studies were case-control studies. The mean age of the participants ranged from 13 to 49 years. Women with known last menstrual period and first trimester dating scan were included (Table 2, Table 3).

**Results on association between periodontitis and preterm birth and/or low birth weight:** The periodontal examination in all of the studies was clinical periodontal status (CPITN, bleeding index, probing pocket depth, and clinical attachment loss). Results from nine case-control studies suggest that periodontal disease is a possible risk factor for adverse pregnancy outcomes [18–26]. Nine studies suggested that periodontal disease is a risk factor for low birth weight [18–21] , preterm birth [22, 23] or preterm low birth weight [24–26]. One study from Tanzania didn't find association between periodontal disease and adverse pregnancy outcomes [27] (Table 2, Table 3).

## Discussion

Periodontal and other oral health problems have been supposed to be a risk factor for preterm birth and /or low birth weight in many literatures. Nevertheless, the association between periodontal health status of a pregnant women and adverse pregnancy outcomes is still controversial. In this review, it has been tried to focus on the results of all case control studies extracted from the literature, which had been conducted to find out the association between periodontal disease and two adverse pregnancy outcomes namely low birth weight and preterm birth. However, the operational definition of Periodontitis differed widely among the selected and reviewed studies since each articles considered special criteria. In this systematic review, nine of the included articles found that periodontal disease is associated with low birth weight and preterm birth [18–26]. Other epidemiological studies which have been done in the past found associations between periodontal status and preterm birth alone (PB) [28] low birth weight (LBW), or preterm birth associated to low birth weight (PLBW) [29]. Even though, the magnitude of the association status varies from adjusted odds ratio of (2.09 to 4.19), this systematic review found that a pregnant woman who is diagnosed as having Periodontitis has a high chance of delivering either a low birth infant or prematurely or both preterm low birth weight infant than a pregnant woman with healthy periodontium. A cohort study done among middle class women of USA found that there was a significant association between having periodontitis and preterm birth and/or low birth weight (A OR 2.26: 95%CI 1.05-4.85) [30]. On the contrary, in this systematic review a single study done in Tanzania found no significant association between periodontal disease and low birth weight or preterm birth and/or PLBW [27]. The reason could be, in this study all subjects were urban dwellers which excludes the rural population that has been reported to have a slightly worse periodontal health compared to urban residents [27]. The reason for controversial evidence could be due to noted potential biases among selected studies. The first and great variation, despite the same methodology, among included studies is that the difference in operational definition of periodontitis. Because there is no universally accepted standard for periodontal disease diagnosis, most of the researchers used their own case definitions (mostly based on disease distribution within the study population) that combined PD and CAL [14]. The additional reason for difference in evidence regarding the adverse outcomes of periodontal disease among pregnant women may be different in maternal socioeconomic status and access to dental care. The majority of the studies done in developing countries, especially those carried out in economically disadvantaged populations who reside in rural settings, suggest that periodontitis is associated with increased risk of preterm birth and low birth weight [10]. We noted potential bias during this systematic review. One of the most challenging potential factors was the variation of periodontal disease definition. Some of the studies used the commonly used clinical measures of periodontal disease (clinical attachment loss and pocket depth). Some studies defined periodontal disease in community periodontal index of treatment needs (CPITN), and bleeding index. The other possible factors that the authors should not forget could be the effect of confounding factors. Though most authors of the included studies tried to control confounding factors there are still questions on the quality of addressing and controlling all confounding factors.

**Limitation of the study:** The authors faced the following limitations during this systematic review; Inconsistent definitions of periodontal disease, and Lack of follow up of the cases in some of the included studies

## Conclusion

This systematic review found that periodontal disease is associated with low birth weight and preterm birth effects. However, more precise studies with proper design, suitable sample size and sufficient follow up period are needed for confirmation. **Practical implications:** this systematic review suggested clinicians to include oral health condition of a pregnant woman with other risk factors in to consideration during antenatal care.

### What is known about this topic

- During pregnancy, immunoresponsiveness and inflammatory response mediators have been changed due to increased progesterone and estrogen levels; this changes result in increased susceptibility of pregnant mothers to periodontal disease.
- Adverse pregnancy outcomes are generally associated with elevated local and systemic inflammatory mediators and intra-uterine infections.

### What this study adds

- This systematic review suggests that there is an association between periodontal disease and preterm and low birth weight. Pregnant mothers who are diagnosed as having periodontal disease may have a high risk of delivering premature and low birth weight child regardless of other confounding factors.
